# F-spondin Is Essential for Maintaining Circadian Rhythms

**DOI:** 10.3389/fncir.2018.00013

**Published:** 2018-02-08

**Authors:** Gabriela L. Carrillo, Jianmin Su, Aboozar Monavarfeshani, Michael A. Fox

**Affiliations:** ^1^Developmental and Translational Neurobiology Center, Virginia Tech Carilion Research Institute, Roanoke, VA, United States; ^2^Graduate Program in Translational Biology, Medicine and Health, Virginia Tech, Blacksburg, VA, United States; ^3^Department of Biological Sciences, Virginia Tech, Blacksburg, VA, United States; ^4^Department of Pediatrics, Virginia Tech Carilion School of Medicine, Roanoke, VA, United States

**Keywords:** suprachiasmatic nucleus, extracellular matrix, circadian rhythm, photoentrainment, intrinsically photosensitive retinal ganglion cells

## Abstract

The suprachiasmatic nucleus (SCN) is the master pacemaker that drives circadian behaviors. SCN neurons have intrinsic, self-sustained rhythmicity that is governed by transcription-translation feedback loops. Intrinsic rhythms within the SCN do not match the day-night cycle and are therefore entrained by light-derived cues. Such cues are transmitted to the SCN by a class of intrinsically photosensitive retinal ganglion cells (ipRGCs). In the present study, we sought to identify how axons from ipRGCs target the SCN. While none of the potential targeting cues identified appeared necessary for retinohypothalamic innervation, we unexpectedly identified a novel role for the extracellular matrix protein F-spondin in circadian behavior. In the absence of F-spondin, mice lost their ability to maintain typical intrinsic rhythmicity. Moreover, F-spondin loss results in the displacement of vasoactive intestinal peptide (VIP)-expressing neurons, a class of neurons that are essential for maintaining rhythmicity among SCN neurons. Thus, this study highlights a novel role for F-spondin in maintaining circadian rhythms.

## Introduction

**Figure 1:**
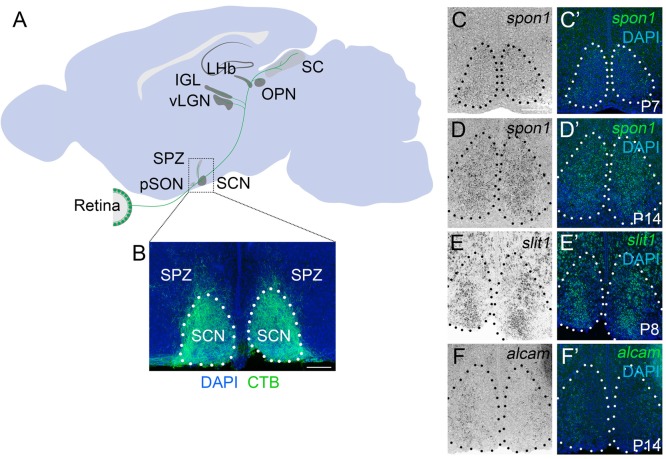
F-spondin,Slit1 and ALCAM are expressed in the developing SCN. **(A)** Schematic representation of the major retino-recipient targets of M1 intrinsically photosensitive retinal ganglion cell (ipRGC) axons (labeled green). SCN, suprachiasmatic nucleus; pSON, peri-supraoptic nucleus; SPZ, subparaventricular zone; vLGN, ventral lateral geniculate nucleus; IGL, intergeniculate leaflet; LHb, lateral habenula; OPN, olivary pretectal nucleus. **(B)** Coronal image of cholera toxin subunit B (CTB)-labeled retinal projections in SCN. Scale bar = 100 μm. **(C–F)** *In situ* hybridization (ISH) for *spon1, slit1* and *alcam* mRNAs in P7–P14 mouse SCN.**(C′–F′)** Depict ISH and DAPI-labeling. Black **(C–F)** and white **(C′–F′)** dots encircle SCN.Scale bar = 100 μm.

Most light-sensitive organisms contain biological clocks that entrain biochemical, physiological and behavioral processes with daily fluctuations of the solar day-night cycle. In mammals, circadian rhythmicity results from the cell-autonomous cyclic transcription and translation of tightly regulated core circadian “clock” genes, including *period* genes (*per1, per2, per3*), *cryptochrome* genes (*cry1* and *cry2*), *brain and muscle arnt-like 1* (*bmal1*) and *circadian locomotor output cycles kaput* (*clock*, Bell-Pedersen et al., 2005; Dibner et al., 2010; Mohawk et al., 2012). The cyclic expression and activity of proteins encoded by these core “clock” genes do not precisely match the 24-h day-night cycle, but rather must be entrained to changes in this cycle by environmental cues. The principle cue that entrains mammalian circadian rhythms to the solar day-night cycle is light. Environmental lighting conditions are detected and transformed into neural activity by retinal photoreceptors and are relayed to a set of densely packed neurons in the suprachiasmatic nucleus (SCN) of the ventral hypothalamus by retinal ganglion cells (RGCs; Figures 1A,B; Fox and Guido, 2011). The SCN then functions as the master regulator of circadian clocks throughout the body and orchestrates the coordination of molecular and biochemical oscillations in all other tissues (Dibner et al., 2010; Mohawk et al., 2012). Neurons within the SCN generate self-sustained oscillations of the core “clock” machinery and intercellular signaling between these neurons, through both synaptic connections and gap junction coupling, allow for the rapid synchronization of light-induced gene expression and oscillations (Challet et al., 2003; Aton and Herzog, 2005). In the absence of light-derived signals, core “clock” machinery within SCN neurons maintain their oscillatory activity but these oscillations “free-run” and fail to entrain to changes in the solar day-night cycle (Foster, 1998; Brzezinski et al., 2005).

Despite the importance of light-derived signals in entraining SCN neurons, only a small subset of RGCs supply photic information to the SCN (Morin and Studholme, 2014). This small set of RGCs express the photopigment melanopsin and are intrinsically photosensitive (Hattar et al., 2002, 2006). Loss of these intrinsically photosensitive RGCs (ipRGCs) leads to “free-running” circadian rhythms even in the presence of a normal solar day-night cycle (Guler et al., 2008). Despite having been under extreme scrutiny since their discovery almost two decades ago, the cellular and molecular mechanisms responsible for regulating ipRGC axon innervation of the SCN remain unclear (Fox and Guido, 2011). In the present study, we sought to answer this question by identifying and testing SCN-specific axonal targeting cues. Using a bio-informatics approach, we identified F-spondin and Slit1, two extracellular matrix proteins, and ALCAM, a cell adhesion molecule, that were all enriched in the adult SCN. The ability of these three cues to direct axonal growth, guidance and targeting in other regions of the developing brain has been well established (Ott et al., 1998; Burstyn-Cohen et al., 1999; Tzarfati-Majar et al., 2001; Plump et al., 2002; Diekmann and Stuermer, 2009). In fact, two of these cues (Slit1 and ALCAM) have established roles in the development of the retinofugal pathway in rodents (Ott et al., 1998; Weiner et al., 2004; Buhusi et al., 2009; Diekmann and Stuermer, 2009). While F-spondin acts as a guidance cue in developing motor circuits (Burstyn-Cohen et al., 1999; Tzarfati-Majar et al., 2001), its role in the developing visual system remains unexplored. It is noteworthy, however, that F-spondin shares homology with the extracellular matrix protein Reelin and can bind canonical Reelin receptors, both of which are important for the assembly of connections between ipRGCs and visual thalamus (Tzarfati-Majar et al., 2001; Hoe et al., 2005; Su et al., 2011, 2013).

Here, we tested whether these factors were necessary for the formation of connections between ipRGCs and the SCN. While our results demonstrate that each of these cues is dispensable for retinohypothalamic targeting, we found that F-spondin-deficient (*spon1^−/−^*) mutants (but not mutants lacking Slit1 or ALCAM) displayed severely disrupted “free-running” rhythmicity. Furthermore, F-spondin-deficient mutants displayed overtly normal circadian rhythms in normal day-night conditions, suggesting that the loss of intrinsic circadian rhythmicity was masked in the presence of light. The rapid ability of *spon1*^−/−^ mutants to adapt to changes in lighting conditions and to entrain to ultradian photoperiods suggests that SCN neurons are weakly coupled in the absence of this extracellular matrix protein. Taken together, these results identify a novel role for F-spondin in maintaining intrinsic circadian rhythms.

## Materials and Methods

### Animals

C57BL/6 mice were obtained from Charles River Laboratories (Wilmington, MA, USA). *Spon1^−/−^* mutant mice were purchased from Taconic Biosciences Inc. (Hudson, NY, USA) and *slit1*^−/−^ were purchased from MMRRC^1^. The generation of *alcam*^−/−^, *math5*^−/−^ and *opn4^taulacz/+^* mice were described previously (Wang et al., 2001; Weiner et al., 2004; Hattar et al., 2006). Genomic DNA was isolated from tail using the HotSHOT method (Truett et al., 2000) and genotyping was performed with the following primers: *spon1* (wildtype, WT) 5′-GAC CGG AGA TCT AGG AAC CCC TAG-3′ and 5′-CAC TCT CGC CAA CAG CTG GAG CG-3′, *spon1* (mutant) 5′-CTC CGC TCA GAG CAG CGC AGC TC-3′ and 5′-CCC TAG GAA TGC TCG TCA AGA-3′; *lacZ* 5′-TTC ACT GGC CGT CGT TTT ACA ACGTCG TGA-3′ and 5′-ATG TGA GCG AGT AAC AAC CCG TCG GAT TCT-3′; *math5*, ATG GCG CTC AGC TAC ATC AT and GGG TCT ACC TGG AGC CTA GC; *neomycin* (neo), GCC GGC CAC AGT CGA TGA ATC and CAT TGA ACA AGA TGG ATT GCA; *slit1* (WT) 5′-AAG ATG CCT CCT CTG ACT TC-3′ and 5′-ACC CTT AGC TTC TAC CAA CC-3′; *slit1* (mutant) 5′-TCT CCT TTG ATC TGA GAC CG-3′ and 5′-AGG TTT CTC GAG CGT CAT AG-3′; *alcam* (common) 5′-AAA GTC GCT GTC CCC CTA AG-3′, *alcam* (mutant) 5′-GGT CTT GTA GTT GCC GTC GT-3′ and *alcam* (WT) 5′-GAG CAG ACC AGT CAA GCC TAA-3′. The following cycling conditions were used on an Eppendorf or Bio-Rad Mastercycler EP: *spon1*, 94°C for 5 min, followed by 33 cycles of amplification (94°C for 30 s, 62°C for 30 s, and 72°C for 45 s) and 10 min at 72°C; *lacZ*, 95°C for 5 min, followed by 35 cycles of amplification (95°C for 30 s, 52°C for 30 s, 72°C for 45 s), and 10 min at 72°C; *math5*, 95°C for 5 min, followed by 35 cycles of amplification (94°C for 30 s, 59°C for 30 s, and 72°C for 45 s) and 10 min at 72°C; *neo*, 94°C for 3 min, followed by 35 cycles of amplification (94°C for 30 s, 56°C for 30 s, and 72°C for 45 s) and 10 min at 72°C; *slit1*, 95°C for 5 min, followed by 30 cycles of amplification (95°C for 30 s, 60°C for 30 s, and 72°C for 30 s) and 10 min at 72°C; *alcam*, 94°C for 2 min, followed by 10 cycles of amplification (94°C for 20 s, 65°C for 15 s and decrease 0.5°C per cycle, 68°C for 10 s) and additional 28 cycles of amplification (94°C for 15 s, 60°C for 15 s, and 72°C for 10 s) and 1 min at 72°C. All analyses conformed to National Institutes of Health (NIH) guidelines and protocols, approved by the Virginia Polytechnic Institute and State University Institutional Animal Care and Use Committees.

### Reagents

All chemicals and reagents were purchased from Fisher (Fairlawn, NJ, USA) or Sigma (St. Louis, MO, USA) unless otherwise stated.

### Immunohistochemistry

Fluorescent immunohistochemistry (IHC) was performed on 16 μm cryosectioned paraformaldehyde (PFA)-fixed brain tissue as described previously (Su et al., 2010, 2012). Briefly, tissue slides were allowed to air dry for 15 min before being incubated with blocking buffer (2.5% normal goat serum, 2.5% bovine serum albumin and 0.1% Triton X-100 in PBS) for 30 min. Rabbit anti-Vasoactive Intestinal Peptide (VIP) antibodies (diluted 1:150 for IHC) were purchased from Immunostar and rabbit anti-vasopressin (AVP) antibodies (diluted 1:1000 for IHC) were purchased from Millipore (Cat# AB1565). Fluorescent secondary antibodies were from Life Technologies (diluted 1:1000). Primary antibodies were diluted in blocking buffer and incubated on tissue sections for overnight at 4°C. On the following day, tissue slides were washed in PBS and secondary antibodies diluted 1:1000 in blocking buffer were applied to slides for 1 h at room temperature. After thoroughly washing in PBS, tissue slides were coverslipped with VectaShield (Vector Laboratories, Burlingame, CA, USA). Images were acquired on a Zeiss Axio Imager A2 fluorescent microscope, a Zeiss Examiner Z1 LSM 710 confocal microscope, or a Zeiss LSM 700 confocal microscope (Oberkochen, Germany). Intensity and area of signal occupation of fluorescent, confocal images were measured in ImageJ as previously described (Singh et al., 2012; Su et al., 2016). A total of 4–6 animals (three sections per animal) were analyzed per genotype and Student’s *T* tests were used to assess statistical significance.

### *In Situ* Hybridization

*In situ* hybridization (ISH) was performed on 16 μm coronal cryosectioned tissues as previously described. Antisense riboprobes were generated from full length cDNAs of *slit1* (MMM1013-98685876), *alcam* (MMM1013-202762192), *spon1* (MMM1013-202701079) and *per1* (MMM1013-202764685; from Open Biosystems; Huntsville, AL, USA). Riboprobes were synthesized using digoxigenin (DIG)-labeled UTP (Roche, Mannheim, Germany) and the MAXIscript *in vitro* Transcription Kit (Ambion, Austin, TX, USA). Probes were hydrolyzed to 500 nt. Coronal brain sections were prepared and hybridized at 65°C as previously described (Su et al., 2010), and bound riboprobes were detected by horseradish peroxidase (POD)-conjugated anti-DIG antibodies and fluorescent staining with Tyramide Signal Amplification (TSA) systems (PerkinElmer, Shelton, CT, USA). Images were obtained on a Zeiss Axio Imager A2 fluorescent microscope or a Zeiss Examiner Z1 LSM 700 confocal microscope. A minimum of three animals per genotype, age and time were compared in ISH experiments.

### Intraocular Injection of Anterograde Tracers

Intraocular injection of cholera toxin subunit B (CTB) conjugated to Alexa Fluor 488 or Alexa Fluor 594 (Invitrogen) was performed in P13 mice as described previously (Su et al., 2011, 2013). After 2 days, mice were killed, and brains were fixed in 4% PFA. One hundred micrometer coronal sections were sectioned on a vibratome (Microm HM 650 V; Thermo Scientific, Waltham, MA, USA) and mounted in VectaShield (Vector Laboratories, Burlingame, CA, USA). Retinal projections in SCN were analyzed from at least three animals for each age and genotype. Images were acquired on a Zeiss Examiner Z1 LSM 710 confocal microscope or a Zeiss LSM 700 confocal microscope.

### Quantitative Real-Time PCR

RNA was isolated from the SCN from both 12:12 LD and DD conditions. Samples from 12:12 light:dark (LD) were isolated at ZT4, ZT10, ZT16 and ZT22. Samples from constant darkness (DD) conditions were isolated at ZT4 5 days after mice were exposed to constant darkness. For samples obtained from the dark phase of ther standard 12:12 LD cycle or from anytime during a DD cycle, mice were euthanized and brain removed in the dark under dim red light illumination. In all cases, brains were removed, vibratomed (200 μm) in iced-cold DEPC-PBS and Suprachiasmatic nuclei (SCN) were dissected. For each sample (at each time point) SCN from a total of six mice was pooled. RNA from these SCN was isolated using the BioRad Total RNA Extraction from Fibrous and Fatty Tissue kit (BioRad). cDNAs were generated from 200 ng RNA with the Superscript II Reverse Transcription First Strand cDNA Synthesis kit (Invitrogen). Quantitative real-time PCR (qPCR) was performed on a Chromo4 Four Color Real-Time system (BioRad) using iQ SYBRGreen Supermix (BioRad; Su et al., 2010). qPCR was performed with the following primers *Bmal1*: 5′-TCCTTCCAGGCAGTCAACTT-3′ and 5′-CTGCAGTGAATGCTTTTGGA-3′; *cry1*: 5′-TGG CAT CAA GAT CCT CAA GA-3′ and 5′-TCC GCT GCG TCT ATA TCC TC-3′; *gjd2*: 5′-TGCTCATCATCGTACACCGT-3′ and 5′-GCAGCAGCACTCCACTATGA-3′; *gapdh*: 5′-CG TCCCGTAGACAAAATGGT-3′ and 5′-TTGATGGCAACA ATCTCCAC-3′; *per1*: 5′-AAC GCT TTG CTT TAG ATC GG-3′ and 5′-TCC TCA ACC GCT TCA GAG AT-3′; *per2*: 5′-GTA TCC ATT CAT GTC GGG CT-3′ and 5′-TAC TGG GAC TAG CGG CTC C-3′; *vip*: 5′-CGT GGT TGT TTT CCT TCG AG-3′ and 5′-GGA GCA GTG AGG GAG ATT CTG-3′. qPCR primers were designed over introns. The following cycling conditions were used with 10 ng RNA: 95°C for 30 s, followed by 40 cycles of amplification (95°C for 5 s, 60°C for 30 s, 55°C for 60 s, read plate) and a melting curve analysis. Relative quantities of RNA were determined using the ∆∆^−CT^ method. A minimum of *n* = 3 experiments (each in triplicate) was run and examined for *spon1*^−/−^ mutants and littermate controls at each time point (ZT4, ZT10, ZT16, ZT22) during 12:12 LD or DD (ZT4). Each individual run included separate *gapdh* control reactions at each time point.

### Wheel Running Activity Assay

Wheel running activity was monitored in wheel cages from Lafayette Instruments which recorded each wheel revolution as an event and sent that information to a compatible computer in 5-min bins using ClockLab software R2011b. All mice were individually housed. Mice were habituated for 2 days before continually recording activity for 2 weeks of 12:12 LD and 2 weeks of DD. To test phase advance and phase delay, activity was recorded for 2 weeks in 12:12 LD, then the onset of light was advanced 6 h and activity was recorded in 12:12 LD. After 2 weeks, the onset of light was delayed 6 h to the original onset of light for 2 weeks of LD. A total of six spon1-mutant and six littermate WT mice were analyzed for the phase advance and delay conditions. To test activity in skeleton photoperiods, *spon1^−/−^* and littermate controls were exposed to 2 weeks of 12:12 LD then were exposed to 2 weeks of 1:11 LD cycle and finally 2 weeks of 12:12 LD. To assess *spon1*^−/−^ and littermate control mice to an ultradian solar cycle, mice were exposed to more than 2 weeks of 12:12 LD circle, then were exposed to 1 week of 3.5 h light/3.5 h dark.

## Results

### Slit1, ALCAM, F-spondin Are Enriched in the Developing Suprachiasmatic Nucleus

To begin to identify potential retinohypothalamic targeting cues we screened available on-line expression databases for any extracellular matrix proteins, growth factors, morphogens or cell adhesion molecules enriched in adult mouse SCN (Lein et al., 2007,^2^). We identified two genes encoding extracellular matrix proteins (Slit1 and F-spondin) and one gene encoding a transmembrane cell adhesion molecule (ALCAM) all of which are enriched in adult SCN. Two of these candidates have previously been reported to contribute to the guidance and growth of retinal axons: members of the Slit family of extracellular matrix proteins mediate retinal pathfinding through binding to Robo receptors (Ringstedt et al., 2000; Plump et al., 2002; Thompson et al., 2006) and the immunoglobulin superfamily adhesion molecule ALCAM (also called BEN, SC1, DM-GRASP, Neurolin and CD166) contributes to the guidance, fasciculation, and topographic mapping of retinal axons (Ott et al., 1998; Weiner et al., 2004; Buhusi et al., 2009; Diekmann and Stuermer, 2009). While F-spondin (which is encoded by the gene *spon1*) has not been implicated in guiding retinal axon growth or subcortical visual circuit formation, it has been implicated in axonal outgrowth in the developing spinal cord (Burstyn-Cohen et al., 1999; Tzarfati-Majar et al., 2001). Importantly, RGCs express amyloid precursor protein (APP), a transmembrane receptor capable of binding to F-spondin (Ho and Südhof, 2004; Osterhout et al., 2015).

Although retinal axons begin to innervate targets in the mouse midbrain during embryogenesis, they do not fully innervate the SCN until the end of the first postnatal week of mouse development (Fox and Guido, 2011; McNeill et al., 2011). To assess whether the expression of these candidate targeting cues coincided with retinohypothalamic innervation, we generated riboprobes and tested whether their mRNAs were present in developing SCN. Not only were all three genes present at early postnatal ages, but each cue exhibited a unique expression pattern in the developing SCN (Figures 1C–F).

### F-spondin, Slit1 and ALCAM Are Not Required for Retinohypothalamic Tract Formation

**Figure 2:**
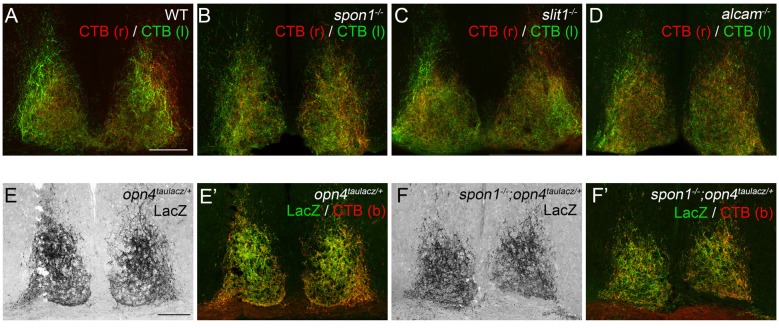
F-spondin, Slit1 and ALCAM are dispensable for retinal innervation of the SCN. **(A–D)** CTB-labeled retinal projections in the SCN of P15 wildtype (WT) **(A)**, *spon1^−/−^* **(B)**, *slit1*^−/−^ **(C)** and *alcam*^−/−^ **(D)** mice. CTB(r)—CTB labeled retinal projections from right eye; CTB(l)—CTB labeled retinal projections from left eye. Scale bar = 100 μm. **(E,F)** M1 ipRGC arbors in the SCN are labeled by immunohistochemistry (IHC) for LacZ in P24 *opn4^tauLacZ/+^* reporter mice. Arbors of M1 ipRGCs correctly target SCN in *spon1*^−/−^; *opn4^tauLacZ/+^* mutant mice **(F)**. **(E′,F′)** show both LacZ-immunoreactivity and CTB-labeling in control **(E′)** and mutant **(F′)** SCN. CTB (b)—binocular CTB labeled retina projections. Scale bar = 100 μm.

Since Slit1, ALCAM and F-spondin are generated in the developing SCN, we assessed their necessity for retinohypothalamic innervation in targeted mouse mutants lacking each cue (i.e., *slit1*^−/−^, *alcam*^−/−^ and *spon1*^−/−^ mutants). All three mutants are viable, fertile, and appear indistinguishable from littermate controls in regards to size and cage activity. To assess retinohypothalamic innervation in these mutants, we performed intraocular injections of fluorescently conjugated CTB, an anterograde tracer that efficiently labels all retinal projections into the brain (Muscat et al., 2003). In controls, retinal projections from each eye project bilaterally to the SCN in each hemisphere on the hypothalamus and these projections arborize to fill the entire SCN (Figure 2A). To our surprise CTB-labeled retinohypothalamic projections appear unaltered by the loss of Slit1, ALCAM or F-spondin (Figures 2B–D).

Since CTB labels all retinal projections indiscriminately, despite only a very small population of RGCs actually projecting axons to the SCN, it remained possible that axons from ipRGCs were no longer innervating SCN in these mutants and instead other classes of RGC axons were present. Consequently, we tested whether ipRGC axons correctly innervated the SCN in mutants lacking F-spondin, the targeting candidate whose developmental expression pattern most closely resembled the distribution of ipRGC arbors in SCN (Figure 1D; Hattar et al., 2006). *Spon1^−/−^* mutant mice were crossed with *opn4^tau-lacz/+^* transgenic mice, in which ipRGC projections are labeled with Tau-LacZ fusion protein (Hattar et al., 2002, 2006). The majority of retinal projections in the SCN of control *opn4^tau-laz/+^* mice contained Tau-LacZ (Figure 2E). Patterns of ipRGC axon arbors in mutants were indistinguishable from controls (Figure 2F). Altogether, our results strongly suggest Slit1, ALCAM and F-spondin are each individually dispensable for the formation of the retinohypothalamic tract.

### F-spondin Deficiency Leads to a Loss of Free Running Rhythmicity

**Figure 3:**
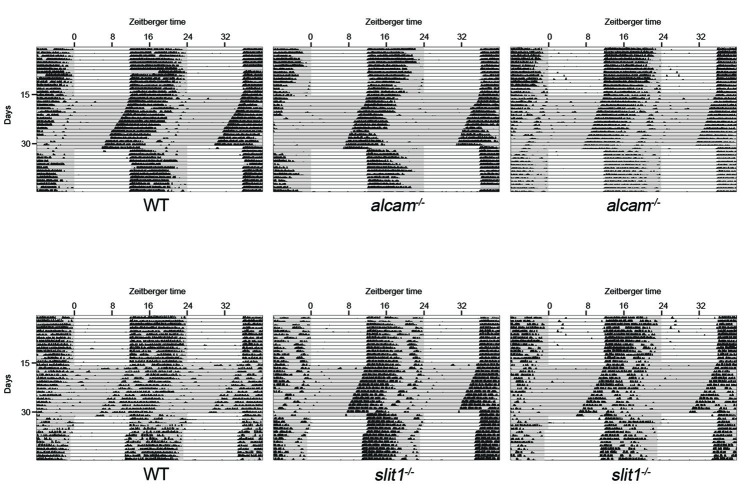
Mice lacking Slit1 and ALCAM exhibit normal photoentrainment and free-running rhythmicity. Representative actograms of wheel running activity of WT, *slit1^−/−^* and *alcam*^−/−^ mice. Mice were housed in a 12:12 light:dark (LD) cycle and then shifted to constant darkness (DD) conditions. Gray background indicates periods of darkness; white background indicates periods of light. Black bars indicate relative level of wheel running activity. Actograms are double plotted.

**Figure 4:**
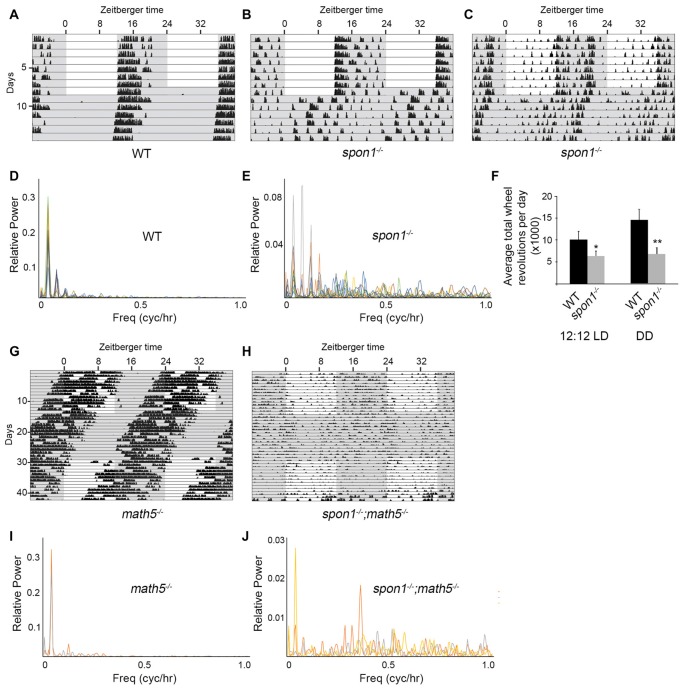
F-spondin deficiency leads to a loss of free running rhythmicity. **(A–C)** Representative actograms of wheel running activity of *spon1^−/−^* mutants **(B,C)** or littermate WT mice **(A)** housed in a 12:12 LD cycle and then shifted to DD conditions. Gray background indicates periods of darkness; white background indicates periods of light. Black bars indicate relative level of wheel running activity. Actograms are double plotted. **(D,E)** Fast Fourier Transform analysis of the activity patterns of 10 WT mice **(D)** and 10 *spon1*^−/−^ mutants **(E)** in DD conditions. Analysis of each individual mouse is shown with a distinct color. **(F)** Levels of wheel running activity of *spon1*^−/−^ mutants was reduced in both 12:12 LD and DD conditions compared with littermate controls. Data are shown as mean ± SEM. *Indicates *p* < 0.05 and **indicates *p* < 0.005 by Students *t*-test (*n* = 14 WT and 27 *spon1*^−/−^ mutants). **(G,H)** Representative actograms of wheel running activity of *math5*^−/−^ mutants **(E)** or *spon1*^−/−^; *math5*^−/−^ mutants **(F)** housed in alternating 12:12 LD and DD cycles. **(I,J)** Fast Fourier Transform analysis of the activity patterns of two *math5*^−/−^ mutant mice **(I)** and three *math5*^−/−^ ; *spon1*^−/−^ double mutants **(J)** in DD conditions. Analysis of each individual mouse is shown with a distinct color.

Given that retinal innervation of the SCN appeared normal in *slit1*^−/−^, *alcam*^−/−^ and *spon1*^−/−^ mutants, we next explored whether these factors may play other roles in SCN function. Since the SCN is the master regulator of circadian rhythms and responsible for the entrainment of these rhythms to changes in light:dark (LD) cycles, we monitored control and mutant mouse activity in normal cycles of 12 h of light and 12 h of darkness (12:12 LD) and in constant darkness (DD). Control mice entrained their activity to 12:12 LD conditions, with a robust increase in activity at the onset of the dark period (Figures 3, 4A). When control mice were shifted to DD, they immediately commenced free-running rhythmicity with each circadian period being less than 24 h in the absence of light signals (period length = 23.63 ± 0.09 [SEM]; *n* = 21; Figures 3, 4A). Actograms of *slit1*^−/−^ and *alcam*^−/−^ mutant activity resembled that of control mice in three ways: (1) their activity was entrained to the dark periods of 12:12 LD (Figure 3); (2) they exhibited free-running rhythmicity in DD (period length for *slit1*^−/−^ mutants = 23.61 ± 0.04 [SEM]; *n* = 8; period length for *alcam*^−/−^ mutants = 23.71 ± 0.04 [SEM]; *n* = 15; Figure 3); (3) their levels of activity in either 12:12 LD or DD appeared comparable to WT controls (Figure 3).

While mice lacking F-spondin also appeared to entrain the majority of their activity to the dark phase of the 12:12 LD cycle (Figures 4A,B), their behavior differed from controls (and from *slit1*^−/−^ and *alcam*^−/−^ mutants) in several ways. First, a cohort of *spon1*^−/−^ mutants exhibited weak, spontaneous activity during the light phase of the 12:12 LD cycle (Figure 4C). Second, their level of activity was significantly reduced compared to controls (Figure 4F), a result that could be explained by previously identified roles for F-spondin in motoneuron and spinal cord development (Burstyn-Cohen et al., 1999; Tzarfati-Majar et al., 2001). Third, and most striking, the majority of mice lacking F-spondin exhibited disrupted free-running rhythmicity in DD conditions (61% of *spon1*^−/−^ mutants exhibited severely disrupted or arrhythmic activity in DD; *n* = 13/21 mutants; Figures 4A–E). These results reveal that *spon1*^−/−^ mutants have major impairment of internal rhythmicity and they suggest that the presence of light is sufficient to mask such impaired activity.

To confirm that *spon1^−/−^* mutants lack rhythmicity in the absence of visual stimuli, we genetically removed retinal inputs to SCN. To accomplish this, we crossed *spon1*^−/−^ mutants to *math5*^−/−^ mutants in which the deletion of the transcription factor Math5 impairs retinogenesis and leads to a failure in RGC development (Wang et al., 2001). *Math5*^−/−^ mutants are viable but lack connections between the retina and retino-recipient nuclei within the brain (Wang et al., 2001; Brooks et al., 2013; Seabrook et al., 2013). For this reason, *math5*^−/−^ mutants are blind and exhibit free-running rhythmicity regardless of the lighting conditions (Figures 4G,I; Brzezinski et al., 2005). Loss of retinal inputs to the SCN in F-spondin-deficient mutants (i.e., *spon1*^−/−^:*math5*^−/−^ double mutants) resulted in arrhythmic locomotor activity regardless of lighting conditions (*n* = 3; all *spon1*^−/−^:*math5*^−/−^ mutants exhibited arrhythmic activity; Figures 4H,J).

### Light Plays a Dominant Influence in Masking the Lack of Internal Rhythms in *Spon1*^−/−^ Mutants

**Figure 5:**
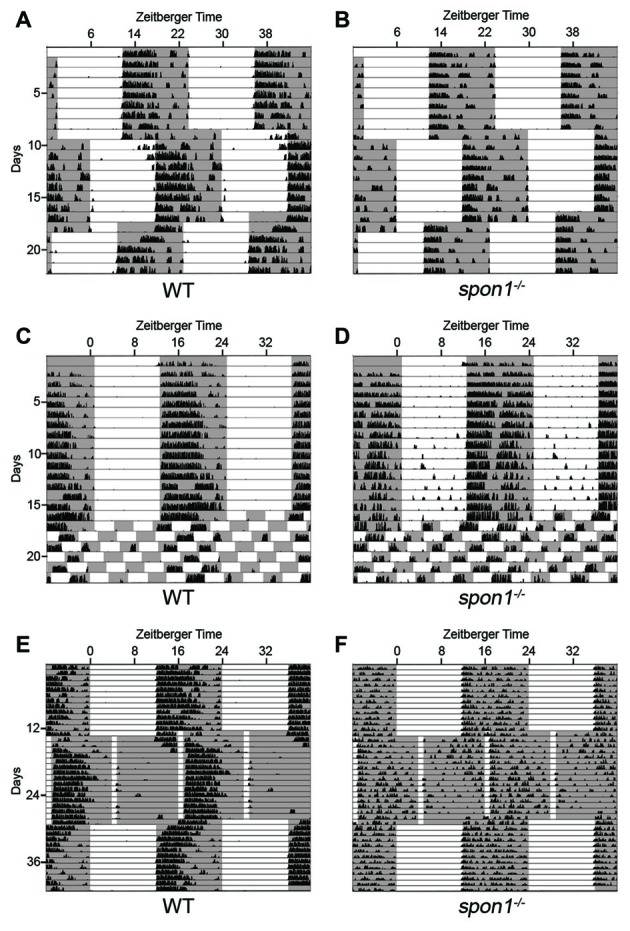
Light masks the lack of internal rhythms in *spon1^−/−^* mutants. **(A,B)** Representative actograms of wheel running activity of *spon1*^−/−^ mutants **(B)** or littermate WT mice **(A)** housed in a 12:12 LD cycle, exposed to a 6 h advance in the light-dark cycle, and then exposed to a 6 h delay in the light-dark cycle. Gray background represents periods of darkness; white background indicates periods of light. Black bars indicate level of wheel running activity. *Spon1*^−/−^ mutants immediately adjust to shifts in the light dark cycle. *n* = 22 WT and 22 *spon1*^−/−^ mutants. **(C,D)** Representative actograms of wheel running activity of *spon1*^−/−^ mutants **(D)** or littermate WT **(C)** housed in a 12:12 LD cycle before being exposed to 7 days of an ultradian light-dark cycle (3.5:3.5 LD). While WT mice cannot entrain to this light-dark cycle, *spon1*^−/−^ mutants exhibit wheel running activity during each dark period of the 3.5:3.5 LD cycle. *n* = 4 WT and eight *spon1*^−/−^ mutants. **(E,F)** Representative actograms of wheel running activity of *spon1*^−/−^ mutants **(D)** or littermate WT **(C)** housed in a 12:12 LD cycle before being exposed to skeleton photoperiod (1:11 LD). While WT mice photoentrain in response to a 1:11 LD cycle, mutants do not. *n* = 5 WT and six *spon1*^−/−^ mutants.

Loss or dysfunction of several core “clock” proteins has been shown to result in arrhythmic behavior in DD, as shown here for a significant number of *spon1^−/−^* mutants (van der Horst et al., 1999; Vitaterna et al., 1999; Bunger et al., 2000; Bae et al., 2001; Zheng et al., 2001). In several cases, it has been demonstrated that the lack of internal rhythmicity and weak synchronization between SCN neurons in these “clock” mutants allow for more rapid adaptation of their activity patterns to changes in lighting conditions (Herzog, 2007; An et al., 2013; Hatori et al., 2014). Therefore, we sought to determine whether the same was true for mutants lacking F-spondin. We assessed behavioral responses to advances or delays in the onset of light in a 12:12 LD cycle in mutant and control mice entrained to a normal 12:12 LD cycle. After 10 days in 12:12 LD the onset of light was advanced 6 h and after an additional 10 days the onset of light was delayed 6 h. It took control mice more than 3 days to re-entrain to the LD cycle that was advanced or delayed by 6 h (Figure 5A). In sharp contrast, *spon1*^−/−^ mutants immediately adapted to shifts in lighting conditions (whether that was an advance or delay in the LD cycle) and began wheel running at the onset of the new dark period (Figure 5B).

These results suggest that light has an immediate and dominant influence on the locomotor activity of *spon1^−/−^* mutants and masks the disrupted of internal rhythmicity in mutants. To further test this, we assessed responses to ultradian lighting conditions, in which the light:dark cycle was reduced to a 7 h cycle (3.5 h light; 3.5 h dark). When shifted from a 12:12 LD cycle to an ultradian LD cycle, WT-mice cannot entrain their circadian rhythmicity to each short photoperiod (Redlin and Mrosovsky, 1999; Redlin et al., 2005; Abraham et al., 2006), and instead display activity only during the dark periods that coincide with dark periods of the previous 12:12 LD cycle (Figure 5C). In contrast, *spon1*^−/−^ mutants exhibited significant bouts of activity during all dark periods and activity was masked in each period of light (Figure 5D). These results support the notion that *spon1*^−/−^ mutants do not actually entrain to 12:12 LD cycles but rather their activity is masked by the presence of light.

Previous studies have demonstrated that short pulses of light coinciding with dusk and dawn are sufficient to maintain circadian rhythms in nocturnal rodents (Schwartz et al., 1996; Aton et al., 2005). If *spon1^−/−^* mutants were not entraining to 12:12 LD cycles, but were merely masking activity in the presence of light, we hypothesized that skeleton photoperiods would be insufficient to entrain mutant activity. To test this hypothesis, we exposed mutant and control mice to a skeleton photoperiod of 1 h of light followed by 11 h of darkness (1:11 LD). In such skeleton photoperiods, WT mice consolidated more than 80% of their daily activity to a singular dark period (Figure 5E; average WT activity in photoperiod 1 = 9676 ± 1388 [SEM] revolutions per cycle and in photoperiod 2 = 2446 ± 725 revolutions per cycle; *p* < 0.01 by Students *t*-test; *n* = 5). In contrast, activity in *spon1*^−/−^ mutants remained disrupted and was less confined to a single photoperiod in this paradigm (Figure 5F; average *spon1*^−/−^ mutant activity in photoperiod 1 = 5893 ± 761 revolutions per cycle and in photoperiod 2 = 4233 ± 451 [SEM] revolutions per cycle; *p* = 0.27 by Students *t*-test; *n* = 6). Thus, short pulses of light appear insufficient to correct impaired internal rhythms in F-spondin-deficient mutants.

### Loss of F-spondin Leads to an Altered Distribution of VIP-expressing Neurons in SCN

**Figure 6:**
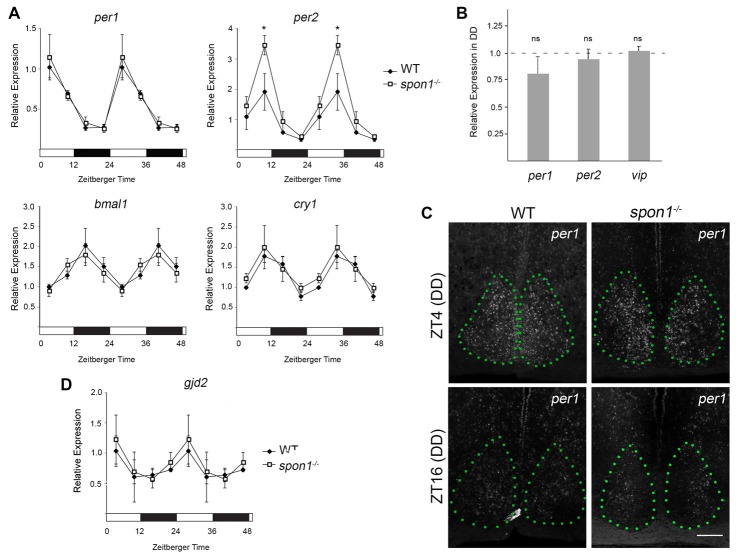
Core clock gene expression in *spon1^−/−^* mutant SCN. **(A)** Expression of *bmal1, cry1, per1* and *per2* mRNAs in the SCN of adult littermate WT mice and *spon1*^−/−^ mutants exposed to 12:12 LD. SCNs were dissected at four time points and mRNA levels were assessed by quantitative real-time PCR (qPCR). Samples were normalized to *gapdh* levels. At ZT4, expression of *per2* was significantly elevated in *spon1*^−/−^ mutants. Data are shown as mean ± SEM. *Indicates *p* < 0.03 by Students *t*-test (*n* = 3 in triplicate per genotype). **(B)** Expression of *per1*, *per2 and vasoactive intestinal peptide (vip)* mRNAs in the SCN of littermate WT mice (dashed line) and *spon1*^−/−^ mutants (gray bars) exposed to 5 days of DD. RNA was isolated at ZT4 and analyzed by qPCR. Samples were normalized to *gapdh* levels. Data are shown as mean ± SEM. ns—not statistically different. **(C)** *ISH* for *per1* mRNA in SCN of adult control and *spon1*^−/−^ exposed to 5 days of DD. Tissue was analyzed at ZT4 and ZT16. SCN depicted by green dots. Scale bar = 100 μm. **(D)** Expression of *gjd2* mRNA in the SCN of WT and *spon1*^−/−^ mutants exposed to 12:12 LD. RNA levels were assessed by qPCR and were normalized to *gapdh* levels. Data are shown as mean ± SEM.

Loss of internal circadian rhythms and immediate arrhythmic activity in DD has been reported in mice lacking core “clock” genes (van der Horst et al., 1999; Vitaterna et al., 1999; Bunger et al., 2000; Bae et al., 2001; Zheng et al., 2001; Abraham et al., 2006), the gap junction protein connexin 36 (Cx36; encoded by the *gjd2* gene; Long et al., 2005), and VIP signaling (Harmar et al., 2002; Aton et al., 2005). We, therefore, tested whether any of these pathways were altered in mutants lacking F-spondin. First, we assessed the expression of *bmal1, cry1, per1* and *per2* mRNAs in mutants and control SCN every 6 h during a normal 12:12 LD cycle. For all genes, the cyclical expression of these circadian genes appeared similar in control and *spon1*^−/−^ SCN (Figure 6A). The only noteworthy difference in circadian gene expression in *spon1*^−/−^ mutants was a significantly elevated expression level of *per2* at the end of the light period (Figure 6A). We also assessed *per1* and *per2* mRNAs in *spon1*^−/−^ mutant and control SCN exposed to DD conditions for 5 days. RNA was isolated at *Zeitbeger time* 4, a time at which controls were likely inactive after 5 days of DD and mutants were likely active (see Figures 2, 3). RNA analysis revealed no significant differences in *per1* or *per2* gene expression after 5 days of DD in the absence of F-spondin (Figure 6B). We confirmed this result by generating riboprobes against *per1* mRNA and performing ISH in the SCN of mutant control mice (Figure 6C). Qualitatively, *per1* mRNA levels and distribution in the SCN appeared similar in mutants and controls at ZT4 and ZT16 after 5 days in DD conditions (Figure 6C). These results were surprising given previous studies on these core clock genes. Based on these results, we interpret this to indicate that disrupted internal rhythms in *spon1*^−/−^ mutants are not the result of loss of core “clock” gene expression in the absence of F-spondin.

Next, we applied similar approaches to address whether *gjd2* mRNA levels were altered in *spon1*^−/−^ SCN. In control mice exposed to 12:12 LD conditions, *gjd2* mRNA levels appeared to be regulated in a rhythmic pattern in SCN, with peak expression coinciding with early phases of the light cycle (similar to *per1* expression patterns; Figure 6D). Similar levels and temporal patterns of *gjd2* were observed in *spon1*^−/−^ mutant SCN (Figure 6D). Taken together, these results suggest that the expression of Cx36 was not perturbed by the loss of F-spondin.

**Figure 7:**
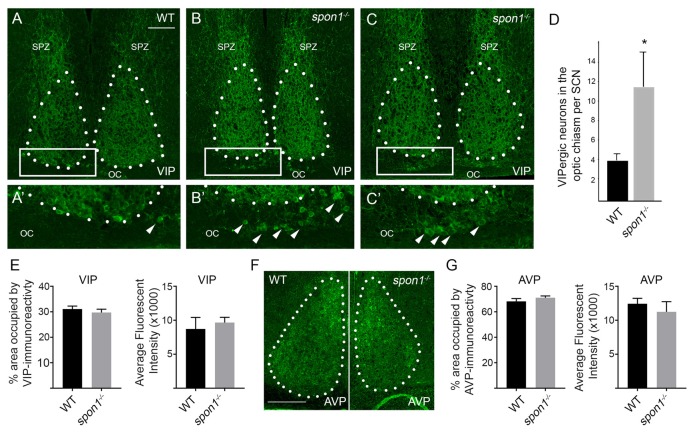
Loss of F-spondin leads to an altered distribution of VIP-expressing neurons in SCN. **(A–C)** IHC for VIP in adult WT and *spon1^−/−^* mutants. Tissue was isolated at ZT4. White dots encircle SCN. Boxed areas are shown in higher magnification in **(A′–C′)**. Arrowheads highlight VIP^+^ neurons in the optic chiasm (OC). SPZ, subparaventricular zone. Scale bar in **(A)** = 100 μm. **(D)** Quantification of VIP^+^ neurons mislocalized in the OC of adult WT and *spon1*^−/−^ mutants. Data are shown as mean ± SEM. *Indicates *p* < 0.03 by Students *t*-test (*n* = 4 per genotype). **(E)** Quantification of the area occupied by VIP-immunoreactivity and the average fluorescent intensity of this signal in the SCN in adult WT and *spon1*^−/−^ mutants. Data are shown as mean ± SEM. **(F)** IHC for anti-vasopressin (AVP) in adult WT and *spon1*^−/−^ mutants. White dots encircle SCN. Scale bar in **(F)** = 100 μm. **(G)** Quantification of the area occupied by AVP-immunoreactivity and the average fluorescent intensity of this signal in the SCN in adult WT and *spon1*^−/−^ mutants. Data are shown as mean ± SEM.

Finally, we addressed whether VIP levels or VIP-expressing neurons were altered by the loss of F-spondin. In WT mice, VIP-containing fibers densely arborize throughout the entire SCN however the majority of VIP-expressing neurons (which receive retinal input) are confined to the ventral most region of SCN that overlies the optic chiasm (OC; Abrahamson and Moore, 2001; Bedont et al., 2014; Fernandez et al., 2016; Figure 7A). In *spon1^−/−^* mutants, we observed similar levels of VIP-immunoreactivity (Figures 7A–C,E) and similar levels of *vip* mRNA (Figure 6B) within the SCN compared to littermate controls. However, an increased number of VIP-expressing neurons were displaced into the OC and optic tract in mutants (Figure 7). In contrast, we detected no difference in the distribution of vasopressin (AVP)-expressing neurons in control or *spon1*^−/−^ mice (Figures 7F,G). Thus, F-spondin appears necessary for the proper migration of VIP^+^ neurons into the SCN.

## Discussion

Although these studies failed to identify targeting cues necessary for guiding ipRGC axons into the SCN, they unexpectedly discovered a novel role for F-spondin in maintaining circadian rhythms in the absence of visual stimuli. Mice lacking *spon1* not only lose intrinsic rhythmicity, but are also more quickly able to modify their activity to changes in the light:dark cycle—results suggesting a loss of neuronal synchrony in the SCN. Although similar results have been observed in mutants lacking core “clock” genes (such as in *cry1*^−/−^:*cry2*^−/−^ double mutants, *cry1*^−/−^:*per2*^−/−^ double mutants, *bmal1*^−/−^ mutants, *per1*^−/−^:*per2*^−/−^ double mutants; van der Horst et al., 1999; Bunger et al., 2000; Bae et al., 2001; Zheng et al., 2001; Oster et al., 2002; Abraham et al., 2006) and gap junctional coupling (*gjd2*^−/−^; Long et al., 2005), we failed to detect significant alterations in the expression or distribution of many of these elements in *spon1*^−/−^. We did detect alterations in the expression of *per2* mRNA in *spon1*^−/−^ mutants in 12:12 LD conditions. Constitutive and dramatic overexpression of Per2 has been shown to abolish intrinsic rhythmicity by increasing nuclear Per2:Cry complexes which inhibits the negative feedback loop of circadian gene transcriptions (Chen et al., 2013; Wang et al., 2016). It seems unlikely that elevated *per2* levels underlie the loss of rhythmicity in *spon1*^−/−^ mutants since the levels are not constitutively upregulated (in either 12:12 LD or DD conditions) and since expression is only modestly upregulated during exposure to light (Figures 4A,B). More striking than these modest increases in *per2* expression, we observed alterations in the distribution of VIPergic neurons in *spon1*^−/−^ mutant SCN. Disruption of VIP signaling (by either the loss of VIP itself, the loss of its receptors, or the loss of transcription factors necessary for the differentiation of VIP neurons) reduces synchrony among SCN neurons and leads to immediate arrhythmic behavior in the absence of visual stimuli (Harmar et al., 2002; Aton et al., 2005; Maywood et al., 2006, 2011; Bedont et al., 2014; Hatori et al., 2014), just as we show here for *spon1*^−/−^ mutants.

VIP neurons comprise 10%–20% of the neurons in SCN (Abrahamson and Moore, 2001). Importantly, VIPergic neurons in the SCN receive direct input from the retina (Fernandez et al., 2016), display induction of activity-dependent immediate early genes (such as cFos) upon delivery of visual stimuli to the retina (Aioun et al., 1998), and are essential for photoentrainment. Although they represent a minority of cells in the SCN, the influence of VIPergic neurons on SCN function is profound with projections that densely arborize throughout the SCN (and adjacent subparaventricular zone [SPZ], a downstream synaptic target of SCN neurons), with the ability to alter the firing rates of more than half the entire population of SCN neurons by releasing VIP (Reed et al., 2001), and with the ability to regulate the coupling of a network of cellular oscillators in the SCN (Aton and Herzog, 2005; Aton et al., 2005). In regards to the studies presented here, it remains unclear whether (*and if so, how*) mislocalization of VIP neurons in *spon1*^−/−^ mutants alters their upstream connectivity with RGCs or downstream connections with other SCN neurons or other targets. Moreover, although F-spondin has known roles in directing cell migration (Debby-Brafman et al., 1999) and the mRNAs of F-spondin receptors are highly expressed in mouse SCN (Allen Brain Atlas; Ho and Südhof, 2004; Hoe et al., 2005; Oka et al., 2015), it remains unclear whether and how F-spondin influences VIPergic neuronal migration in the SCN. Moreover, while we identified F-spondin as being dramatically enriched in SCN vs. other brain regions, it is generated in other brain regions (Allen Brain Atlas). It remains possible that its expression and function in other brain regions (especially those associated with circadian behaviors, such as the intergeniculate nucleus) contribute to alterations in circadian behaviors observed here.

While this study is the first to demonstrate a role for F-spondin in maintaining intrinsic rhythmicity and circadian function, there are in fact few known roles for this ECM molecule in the brain. A recent series of studies, however, has identified *spon1* gene variants and risk alleles in influencing dementia severity and cognitive decline (Jahanshad et al., 2013; Sherva et al., 2014; Varol et al., 2017). Loss or mutation of F-spondin may affect dementia severity via its role in binding APP and clearing Aβ peptides (Ho and Südhof, 2004; Habisch et al., 2010). However, in light of results presented here, it is also possible that F-spondin’s role in maintaining circadian rhythms contribute to limiting AB pathology and pathogenesis of Alzheimer’s disease (AD). Disruption of circadian rhythms have well established roles in the pathogenesis of AD (Musiek, 2015)—roles that are both consequences of the disease and potentially causative of the disease. In support of the latter, mutations in core “clock” genes have been identified (like those in *spon1*) that are associated with increased risk for patients developing AD (Chen et al., 2013). With such studies in mind, it is possible that disruption of intrinsic rhythms in F-spondin-deficient mammals may have significant impact on the development and progression of cognitive and neurodegenerative diseases.

## Author Contributions

GLC, JS and AM carried out all of the collection, preparation and analysis of data for these studies. Specifically, GLC performed bioinformatics screening, behavioral analysis and analysis in *spon1^−/−^* mutants. JS and AM performed ISH and analysis of *slit1*^−/−^ and *alcam*^−/−^ mutants. GLC and JS helped draft portions of the manuscript. MAF helped conceive, design and coordinate the study and drafted the manuscript. All authors read and approved the final version of the manuscript.

## Conflict of Interest Statement

The authors declare that the research was conducted in the absence of any commercial or financial relationships that could be construed as a potential conflict of interest.

## Footnotes

^1^https://www.mmrrc.org

^2^http://mouse.brain-map.org
